# The Book World of Medicine and Science

**Published:** 1900-04-14

**Authors:** 


### The Book World of Medicine and Science.

System of Diseases of the Eye. By American, British, Dutch, French, German, and Spanish Authors. Edited by William F. Norris, A.M., M.D., and Charles A. Oliver, A.M., M.D. of Philadelphia, Pa., U.S.A. Volume IV.: Motor Apparatus, Cornea, Lens, Refraction, Medical Ophthalmology. With 51 full-page plates and 211 text illustrations. (Philadelphia and London: J. B. Lippincott Company. 1900. 21s. net.)

This volume completes the most extensive work on eye disease which has yet been written in the English language, and we are now able to judge of it as a whole. It seems to us a pity that when this great work was being planned, seeing that it was to be written in English, it was not thought desirable that it should be written entirely by English-speaking ophthalmic surgeons. In the present volume out of sixteen articles no less than eight are translations. The difficulty of editing a work of this magnitude with so many different authors must be considerable, and we heartily congratulate Drs. Norris and Oliver on the very successful completion of their labours; at the same time, we must say that the bulk of the several volumes could, with advantage, have been considerably reduced if they had been more careful to prevent repetition and overlapping. To take but one of the numerous examples which might be quoted; we have in this volume an article on diseases of the lens by one of the editors himself—Dr. W. F. Norris—in which he enters very fully into its congenital abnormalities, notwithstanding that the same conditions had also been fully dealt with in the first volume in an article specially devoted to congenital malformations. This volume opens with a very thorough article by Dr. Landolt, of Paris, on that special branch of his speciality to which he has devoted so much attention, the anomalies of the motor apparatus of the eyes. Dr. J. P. Nuel, of Liége, writes on diseases of the cornea. Having first treated of inflammation of the cornea generally, he divides his subject up under the following headings: (*a*) "Superficial Keratitis"; (*b*) "Deep Keratitis"; (*c*) "Sequelæ and Consequences of Keratitis"; (*d*) "Neoplasms and Tumours of the Cornea; Corneal Leprosy." One omission we note, that is, any reference to the peculiar condition of cornea sometimes met with, due to dissemination of blood pigment through it. Dr. W. F. Norris' article on the lens occupies 143 pages; he enters very extensively into the pathology of the various forms of cataract. It is illustrated by a number of very well reproduced photo-micrographs. Dr. C. A. Oliver contributes a very wordy chapter on "Ametropia: Its Etiology, Course, and Treatment," the character of which may be judged by quoting a few of the headings: "The Effect of the Eye on Mentality," "Dynamic Exposure of Hypermetropia," "Significance of Therapy on Ametropia," "Loose-Lens Selection." Professor Haab's article on "Ocular Lesions Dependent upon Diseases in the Circulatory System"; Mr. Swanzy's on "Eye Disease and Eye Symptoms in Relation to Organic Diseases of the Brain and Spinal Cord"; Mr. Lawford's on "Ocular Lesions Dependent upon Disorders of the Secretory and Excretory Organs"; and Mr. J. B. Story's on "Ocular Lesions in Variola, Rubiola, Morbilli, Scarletina, Erysipelas, and Diphtheritis," are all ably written, well up to date, and leave little to be desired. Dr. Santos-Fernandez's chapter on the ocular lesions of influenza, dysentery, cholera, malarial fever, dengue, and yellow fever leaves us with the impression that there is here still a large field for accurate clinical observation. The ocular manifestations of hysteria, Dr Parinand, of Paris, treats under two headings: Hysterical amblyopia and hysterical affections of the motor apparatus of the eyes. He would, we think, have done well to have dealt more fully with the differential diagnosis of the former affection. The eye affections due to Graves's disease and herpes zoster are briefly described by Mr. Jonathan Hutchinson, jun. He dismisses the operation of removal of a portion of the thyroid gland in extreme cases of exophthalmos in a few words, and says nothing as to the operation of division of the cervical sympathetic. Dr. Myles Standish writes on the much-discussed question of the motor changes in the ocular apparatus associated with functional neuroses. He concludes "it is too early as yet to estimate even approximately the relative importance of ocular strain in the production of functional nervous disturbances;" and "that the proportion is small will be conceded by all fair minded observers." The article on toxic amblyopias by Dr. De Schweinitz is complete and concise. The entozoa of the human eye are described by Dr. Maximilian Salzmann, of Vienna; simulated blindness and the various means employed for its detection, by Dr. Baudry, of Lille; the system very fitly concluding with an article on the ocular signs of death by Dr. Gayet, of Lyons.

